# Off-the-Shelf Partial HLA Matching SARS-CoV-2 Antigen Specific T Cell Therapy: A New Possibility for COVID-19 Treatment

**DOI:** 10.3389/fimmu.2021.751869

**Published:** 2021-12-23

**Authors:** Nayoun Kim, Jong-Min Lee, Eun-Jee Oh, Dong Wook Jekarl, Dong-Gun Lee, Keon-Il Im, Seok-Goo Cho

**Affiliations:** ^1^ Product Development Division, LucasBio Co., Ltd., Seoul, South Korea; ^2^ Division of Respiratory, Allergy and Critical Care Medicine, Department of Internal Medicine, Seoul St. Mary’s Hospital, College of Medicine, The Catholic University of Korea, Seoul, South Korea; ^3^ Department of Laboratory Medicine, Seoul St. Mary’s Hospital, College of Medicine, The Catholic University of Korea, Seoul, South Korea; ^4^ Division of Infectious Diseases, Department of Internal Medicine, College of Medicine, The Catholic University of Korea, Seoul, South Korea; ^5^ Institute for Translational Research and Molecular Imaging, The Catholic University of Korea, Seoul, South Korea

**Keywords:** COVID19, T cell therapy, HLA sharing, virus specific T cells, viral immunity

## Abstract

**Background:**

Immunological characteristics of COVID-19 show pathological hyperinflammation associated with lymphopenia and dysfunctional T cell responses. These features provide a rationale for restoring functional T cell immunity in COVID-19 patients by adoptive transfer of SARS-CoV-2 specific T cells.

**Methods:**

To generate SARS-CoV-2 specific T cells, we isolated peripheral blood mononuclear cells from 7 COVID-19 recovered and 13 unexposed donors. Consequently, we stimulated cells with SARS-CoV-2 peptide mixtures covering spike, membrane and nucleocapsid proteins. Then, we culture expanded cells with IL-2 for 21 days. We assessed immunophenotypes, cytokine profiles, antigen specificity of the final cell products.

**Results:**

Our results show that SARS-CoV-2 specific T cells could be expanded in both COVID-19 recovered and unexposed groups. Immunophenotypes were similar in both groups showing CD4+ T cell dominance, but CD8+ and CD3+CD56+ T cells were also present. Antigen specificity was determined by ELISPOT, intracellular cytokine assay, and cytotoxicity assays. One out of 14 individuals who were previously unexposed to SARS-CoV-2 failed to show antigen specificity. Moreover, ex-vivo expanded SARS-CoV-2 specific T cells mainly consisted of central and effector memory subsets with reduced alloreactivity against HLA-unmatched cells suggesting the possibility for the development of third-party partial HLA-matching products.

**Discussion:**

In conclusion, our findings show that SARS-CoV-2 specific T cell can be readily expanded from both COVID-19 and unexposed individuals and can therefore be manufactured as a biopharmaceutical product to treat severe COVID-19 patients.

**One Sentence Summary:**

Ex-vivo expanded SARS-CoV-2 antigen specific T cells developed as third-party partial HLA-matching products may be a promising approach for treating severe COVID-19 patients that do not respond to previous treatment options.

## Introduction

Coronavirus disease 2019 (COVID-19), caused by the novel severe acute respiratory syndrome coronavirus-2 (SARS-CoV-2), has caused significant medical, social, and economic disruptions worldwide. The recent development of vaccines by global pharmaceutical companies has brought us a step closer to eradicating the virus. However, while vaccination can prevent future infections, there have already been more than 3,025,835 deaths due to the absence of appropriate therapeutic measures, and this number continues to increase (1). Approximately 20% of COVID-19 patients develop life-threatening pneumonia, which requires extensive medical care. An increasing number of cases, along with a limited number of available intensive care units and medical teams, has led to a collapse of healthcare systems in many countries (2).

In this study, we investigated the role of T cell responses in viral immunity and hypothesized that adoptive T cell therapy may be a promising and safe approach to combat SARS-CoV-2. Virus-specific T cells (VST) have been used in the treatment of various infectious diseases (3). However, the majority of VST therapies have been focused on latent DNA-virus infections, especially in the hematopoietic stem cell transplantation setting (4, 5), instead of acute infections. COVID-19 is a challenging clinical situation for VST application. COVID-19 is an acute viral infection associated with pro-inflammatory cytokine storms that may negatively impact the infused T cells. Moreover, T cells are more readily expanded in convalescent donors in the presence of memory T cells and, therefore, the donor-sources for SARS-CoV-2-specific T cells may be limited. It has been reported, however, that unexposed healthy individuals contain cross-reactive T cells against common coronaviruses that may respond to SARS-CoV-2 (6, 7). Researchers have already attempted to use VSTs to treat high-risk COVID19 patients in the absence of other effective treatments. These studies have adopted various T-cell production methods, including culture-expansion (8, 9) and automatic selection methods (10, 11). Regardless of the method used, CD4+ T cells, especially type 1 helper T (Th1) cells, dominated over CD8+ T cells in SARS-CoV-2 specific T-cell immunity (6, 7, 12).

In this study, we aimed to demonstrate the effects of culture-expanded SARS-CoV-2-specific T cells from both COVID-19 recovered and unexposed individuals. We demonstrated that these ex-vivo expanded cells were cytotoxic to SARS-CoV-2 protein-expressing target cells and may also exhibit immunosuppressive properties to inhibit pro-inflammatory cytokines. We suggest two different clinical applications of these cells, either as personalized autologous vaccines or as third-party off-the-shelf bioproducts.

## Materials And Methods

### Donors

Seven recovered COVID-19 patients, who were treated at Seoul St. Mary’s Hospital, were recruited for blood donations. Thirteen randomly selected blood donors, unexposed to SARS-CoV-2, were also recruited. All donors fulfilled the blood bank eligibility criteria and were examined for clinical signs of COVID-19, including fever and respiratory symptoms, in accordance with the hospital’s quarantine regulations. All donors signed consent forms approved by the Institutional Review Board of Seoul St. Mary’s Hospital, and all procedures were performed in accordance with the Declaration of Helsinki (KC20TSSI0274, KC20TSSI0872).

For recovered donors, individuals who have been quarantined after confirmation of COIVD-19 or who have received treatment and met all the release criteria from public quarantine was defined as recovered. Clinically, individuals did not show fever and showed improvement in clinical symptoms even without taking antipyretic drugs. Furthermore, recruited individuals must have had confirmed negative PCR test results at least twice at 24-hour intervals. For unexposed donors, individuals must have no fever or respiratory symptoms and must be SARS-CoV-2 Ab IgG antibody negative. For both recovered and unexposed donors the exclusion criteria include those who are HIV antibody positive, syphilis positive, hepatitis B or C carriers or actively infected with other serious infections requiring medical treatment.

We obtained either 50 cc of whole blood or one blood volume of leukapheresis from each donor. This study was approved by the Clinical Research Information Service, Republic of Korea, and registered in the World Health Organization Registry Network (KCT0005370, KCT0005864). Donor characteristics are shown in **Table 1**.

**Table 1:** Donor characteristics.

Donor No.	COVID19	Sex/Age	SARS-CoV2IgG/IgM^*^	COVID19 severity	COVID19 treatment used	Days to recovery from diagnosis	Days from recovery to cell production
1	Recovered	F/61	3+/1+	Mild	Steroids, Haloxin, Kaletra	23	103
2	Recovered	M/61	4+/1+	Severe	Steroids, Haloxin, Kaletra	31	90
3	Recovered	M/43	1+/-	Mild	Steroids, Haloxin, Kaletra	14	113
4	Unexposed	M/29	-/-	N.A	N.A	N.A	N.A
5	Unexposed	F/33	-/-	N.A	N.A	N.A	N.A
6	Unexposed	F/27	-/-	N.A	N.A	N.A	N.A
7	Unexposed	F/23	-/-	N.A	N.A	N.A	N.A
8	Unexposed	M/33	-/-	N.A	N.A	N.A	N.A
9	Unexposed	F/30	-/-	N.A	N.A	N.A	N.A
10	Unexposed	M/32	-/-	N.A	N.A	N.A	N.A
11	Unexposed	F/27	-/-	N.A	N.A	N.A	N.A
12	Unexposed	F/27	-/-	N.A	N.A	N.A	N.A
13	Unexposed	M/25	-/-	N.A	N.A	N.A	N.A
14	Recovered	F/58	N.T.	Mild	Steroids, Haloxin, Kaletra	26	87
15	Recovered	F/60	4+/-	Mild	Steroids	15	78
16	Recovered	M/23	-/+	Mild	None	Unknown	Unknown
17	Recovered	M/23	2+/-	Mild	None	Unknown	Unknown
18	Unexposed	F/29	-/-	N.A	N.A	N.A	N.A
19	Unexposed	F/64	-/-	N.A	N.A	N.A	N.A
20	Unexposed	F/39	-/-	N.A	N.A	N.A	N.A

N.A, not applicable; N.T, not tested.

^*)^ Test results from SGTi-flex COVID-19 IgM/IgG assay (Sugentech Inc., Korea) were used.

### Human Leukocyte Antigen (HLA) Testing

For HLA typing of donors, an aliquot of the peripheral blood was sent to the Catholic Hematopoietic Stem Cell Bank for analysis. The sequence-based typing (SBT) method was used as previously described (13). DNA was extracted from whole blood in ethylenediaminetetraacetic acid (EDTA)-coated blood containers. The AlleleSEQR kit (Abbott, Chicago, IL, USA) was used for HLA Class I and Class II genotyping, according to the manufacturer’s instructions. Sequencing was performed using the ABI 3130XL genetic analyzer (Applied Biosystems, Waltham, MA, USA) with POP 6 polymer for exons 2, 3, and 4 of HLA-A, B, and C, and for exon 2 and codon 86 for HLA-DRB1. HLA types were analyzed using SBTengine (Genome Diagnostics B.V., Utrecht, Netherlands), and reanalyzed using Assign SBT v3.5 (Conexio Genomics, Applecross, Australia). The HLA types of donors are listed in **Table 2**.

**Table 2:** HLA types of Donors.

Donor No.	HLA-A		HLA-B		HLA-C		HLA-DRB	
1	02:01	33:03	55:02	58:01	03:02	03:03	12:01	13:02
2	24:02	26:02	15:07	51:02	03:03	15:02	04:03	09:01
3	11:01	24:02	54:01	54:01	01:02	01:02	04:05	08:03
4	02:01	33:03	15:07	58:01	03:02	03:03	04:03	13:02
5	24:02	30:01	07:02	40:06	07:02	08:01	01:01	08:03
6	24:02	30:01	13:02	40:02	03:04	06:02	07:01	11:01
7	24:02	26:02	51:01	54:01	07:02	14:02	11:01	14:05
8	02:01	02:01	07:02	15:11	03:03	07:02	09:01	15:01
9	24:02	33:03	40:06	44:03	01:02	14:03	09:01	13:02
10	02:06	33:03	44:03	54:01	01:02	14:03	13:02	15:01
11	02:07	24:02	15:11	46:01	01:02	03:03	09:01	09:01
12	02:01	24:02	27:05	40:06	01:02	02:02	14:05	15:02
13	24:02	24:02	15:07	56:01	01:02	03:03	04:03	14:54
14	02:01	11:01	51:01	15:11	03:03	15:02	04:05	14:05
15	02:01	30:01	40:06	44:03	03:03	07:06	0701	14:03
16	24:02	33:03	35:01	44:03	03:04	07:06	04:03	07:01
17	02:01	03:01	13:01	27:05	02:02	03:04	04:05	15:02
18	02:06	30:01	13:02	51:01	06:01	14:02	04:05	11:01
19	24:02	24:01	51:01	54:01	01:02	14:02	04:05	12:01
20	24:02	33:03	44:03	51:01	07:06	14:02	07:01	12:01

### Anti-SARS-CoV-2 IgG and IgM Detection

We used different commercial assays according to the manufacturer’s instructions to detect Anti-SARS-CoV-2 IgG and IgM. We used SGTi-flex COVID-19 IgM/IgG assay (Sugentech Inc., Korea), SARS-CoV-2 IgG assay (Abbott, Chicago, IL, USA), Elecsys Anti-SARS-CoV-2 assay (Roche Diagnostics, Basel, Switzerland), and ADVIA Centaur SARS-CoV-2 Total assay (Siemens, Munich, Germany).

### SARS-CoV-2 Specific T Cell (SARS-CoV-2 Specific CTL) Generation

To generate SARS-CoV-2 specific CTLs, peripheral blood mononuclear cells (PBMCs) were isolated using Ficoll gradient centrifugation. Then, at least 1x10^6^ PBMCs were seeded in an appropriate well plate or flask, depending on the starting cell numbers, at a cell density of 1x10^7^ cells/mL. PBMCs were stimulated with Peptivators for SARS-COV-2 Spike (S), Membrane (M), and Nucleocapsid (N) (1 ug/mL; Miltenyi Biotec, Bergisch Gladbach, Germany) proteins. Peptivator is a pool of lypophilized peptides, consisting of 15-mer sequences with 11 amino acids overlap, covering the entire sequence of each protein. Peptivator for Spike protein only covers the immunodominant sequence domains of the spike glycoprotein. On the same day, the cells were further stimulated with 50ng/mL of recombinant human interferon-gamma (rhIFN-γ; R&D systems, Minneapolis, MN, USA). A few days later, the cells were expanded using 60ng/mL of recombinant human interleukin-2 (rhIL-2; R&D systems), applied every 3–4 days for 3–4 weeks. Cells were suspended and maintained in 5% human serum (Sigma-Aldrich, St. Louis, MO, USA) containing AIM-V medium (Gibco; Thermo Fisher Scientific, Wilmington, DE, USA) at 37°C under 5% (v/v) CO_2_. Cells were later harvested and cryopreserved for further characterization.

Six out of twenty leukapheresis products were produced at the cell processing facility of the Catholic Institute of Cell Therapy, at the Catholic University of Korea, in accordance with good manufacturing practices (GMPs). The same manufacturing method was performed using animal-free reagents. On the day of harvest, phenotypes and functional potencies of the cells were characterized. The final products were also tested for sterility, mycoplasma, endotoxins and adventitious viruses.

### Preparation of PHA-Blasts

Phytohaemagglutinin (PHA)-induced blasts were prepared from autologous PBMCs stimulated with PHA (3 μg/mL; Sigma-Aldrich) and rhIL-2 (25 IU/mL) in 5% human serum containing AIM-V medium for 3–4 days. For peptide pulsing, PHA-blasts (1x10^7^/mL) were harvested and incubated with CMV pp65 peptivator (Miltenyi Biotec) at a concentration of 2 μg/mL and incubated at 37°C, under 5% (v/v) CO_2,_ for 2 hours.

### Immunophenotyping

Immunophenotyping of PBMCs was performed by staining for various surface-marker combinations using the following fluorescence-conjugated antibodies: CCR7 (3D12), CD3 (UCHT1), CD4 (SK3), CD8 (SK1), CD14(61D3), CD16 (CB16), CD19 (HIB19), CD45RA (H100), CD45RO (UCHL1), CD56 (TULY56, CMSSB), CD57 (QA17A04; Biolegend, San Diego, CA, USA), CD62L (DREG-56), Lag3 (3DS223H), PD-1 (EH-12.2H7; Biolegend), and Tim3 (F38-2E2). All antibodies were purchased from eBioscience, Inc. (San Diego, CA, USA), unless mentioned otherwise. Cells were washed once with flow cytometry staining buffer, pelleted, and antibodies were added prior to incubation in the dark, at room temperature for 15 minutes. The cells were then washed and analyzed. Flow cytometry gating strategy for various lymphocytes were first based on a low forward scatter (FSC) and low side scatter (SSC) gating of lymphocytes. Then, T cell markers CD3, CD4, CD8; NK cell marker CD56 were used to identify the major lymphocyte subsets. Various markers were used to assess activation, cytokine production, and exhaustion.

### Intracellular Cytokine Staining Following Peptide Stimulation

Expanded cells were stimulated overnight with SARS-COV-2 S, M, and N peptivators (1 μg/mL each; Miltenyi Biotec). Protein transport inhibitor containing monensin (BD GolgiStop; Pharmingen, San Diego, CA, USA) was added in the last 4h of incubation at 37°C, under 5% (v/v) CO_2_. Positive controls were stimulated with cell stimulation cocktails containing phorbol 12-myristate 13-acetate (PMA), ionomycin, brefeldin A, and monensin (Invitrogen Corp.; Waltham, MA, USA) while negative controls were not peptide-stimulated. Cells were first stained for surface markers: CD3, CD56, CD8, and CD4. Intracellular IFN-γ and TNF-α, IL-2 staining was performed according to the manufacturer’s instructions for the kit (eBioscience). Flow cytometry was performed using the BD Biosciences Fortessa cytometer.

### Activation-Induced Marker (AIM) Assay

Expanded cells were stimulated with SARS-COV-2 S, M, and N peptivator (1 μg/mL each; Miltenyi Biotec). Unstimulated cells were used as negative controls. Following overnight stimulation, cells were surface stained for CD3, CD4, CD8, CD56, CD137 (4B4), CD154 (14–21), CD25 (BC96), CD38 (HIT2), CD69 (FN50), and HLA-DR (L243). Flow cytometry was performed using the BD Biosciences Fortessa cytometer.

### Foxp3 Staining for Treg Cell Detection

To detect Treg cells, Foxp3 staining was performed using the eBioscience Foxp3 staining kit. Expanded cells were stained for surface markers: CD3, CD25, CD127 (EBioRDR5; eBioscience), CD4, and CD8. The cells were resuspended in 500 μL fixation/permeabilization buffer and incubated for 30 minutes at 4°C, and then washed in permeabilization buffer. The cells were then pelleted and incubated with FoxP3 (PCH10; eBioscience) antibodies for 30 minutes at 4°C. Flow cytometry was performed using the BD Biosciences Fortessa cytometer. In some experiments, the cells were stimulated with peptides overnight, prior to Foxp3 staining.

### Cell Proliferation Assay

To measure alloreactivity and cell proliferation, expanded CTLs were labeled with CellTrace Cell Proliferation kit (Invitrogen) according to the manufacturer’s instructions. The SARS-CoV-2-specific CTLs were incubated with CellTrace CFSE dye for 20 minutes at room temperature in the dark. Cells were then washed with cell culture medium to remove remaining free dye from the solution. Labeled cells were pelleted by centrifugation and resuspended in cell culture medium, and then incubated for at least 10 minutes before cell stimulation. Carboxyfluorescein diacetate succinimidyl ester (CFSE) labelled SARS-CoV-2-specific CTLs were co-cultured for 5 days with allogeneic PBMCs or non-CD4, HLA-DR+ sorted antigen presenting cells. Acquisition and analysis were completed using the FACS Canto cytometer (BD Biosciences).

### Cytotoxicity *Assay*

Flow cytometry-based cytotoxicity assay was performed as previously described (22). The target cells (1x10^6^/mL) were labeled with CFSE in complete culture medium at room temperature, under 5% CO_2_ for 20 minutes, as per the manufacturer’s protocols. Target cells (1x10^5^) were incubated overnight with the effector cells at different effector:target ratios. Prior to flow cytometric acquisition, cells were stained with 7-Aminoactinomycin D (7-AAD; eBioscience) and cytotoxicity was evaluated using a FACS Canto cytometer (BD Biosciences). Target cells were gated on CFSE+ cells and then examined for cell death by uptake of 7-AAD. The percentage of effector cell mediated cytotoxicity was then calculated using the following equation:


$$Cytotoxicity\;(\%)=\frac{(\text{Dead target cells}(\%)-\text{spontaneous deaths }(\%))\times 100}{\left(100-\text{spontaneous deaths }(\%)\right)}$$

### ELISPOT Assay

For detection of IFN-γ secreting cells, ELISPOT assays were performed using the BD ELISPOT assay kit, according to the manufacturer’s instructions. The final T cell products were serially diluted from 1x10^5^ to 1.25x10^4^ cells/well. SARS-CoV-2 antigen specific activity was measured using SARS-CoV-2 S, M, N peptivators (1 μg/mL each; Miltenyi Biotec). Each culture condition was run in triplicate. The number of spots corresponding to IFN-γ secreting cells was counted using an AID-ELISPOT-Reader.

### Luminex Multiplex Cytokine Assay

Concentrations of the following immune molecules were determined using a magnetic bead-based immunoassay: CD40L, CD137 (4-1BB), CD152 (CTLA4), GM-CSF, Granzyme B, ICAM-1, IFN-α, IFN-β, IFN-γ, IL-1β, IL-1RA, IL-2, IL-2R, IL-4, IL-6, 7, IL-8 (CXCL8), IL-9, IL-10, IL-12p70, IL-15, IL-17A, IL-21, IL-23, perforin, and TNF-α (Procartaplex; Thermo Fisher Scientific, Wilmington, DE, USA). Cell culture supernatant samples were obtained from SARS-CoV-2-specific CTLs before and after SARS-CoV-2 peptide stimulation. The median fluorescence intensities of analytes were detected using the flow-based MAGPIX System (MilliporeSigma, Burlington, MA, USA). Cytokine concentrations were calculated using Luminex xPONENT v. 4.2 software, using a standard curve derived from known reference concentrations supplied by the manufacturer. A five-parameter model was used to interpolate the final concentrations. Values were expressed as pg/mL.

### Data Analysis

Flow cytometry data were analyzed using FlowJo 9.9.6 (FlowJo, LLC., Ashland, OR, USA). Prism (GraphPad Software Inc., San Diego, CA, USA) was used for plotting data and statistical analyses. Data were presented as means ± ranges, unless indicated otherwise. The number of donors was denoted by *n*.

### Statistical Analysis

Data were presented as means ± standard deviations (SDs) unless indicated. Mann–Whitney U test or Student’s t-test were used for comparison between two groups, while the Kruskal–Wallis test was used for comparison between multiple groups. Statistical analyses were performed using the SPSS Statistics software (version 16.0; IBM Inc., Armonk, NY, USA). P-values < .05 were considered significant.

## Results

### Dominant CD4+ Phenotypes in *Ex Vivo* Culture-Expanded SARS-CoV-2 Specific T Cells Mainly Consisting of Effector Memory Cells

**Figure 1:**
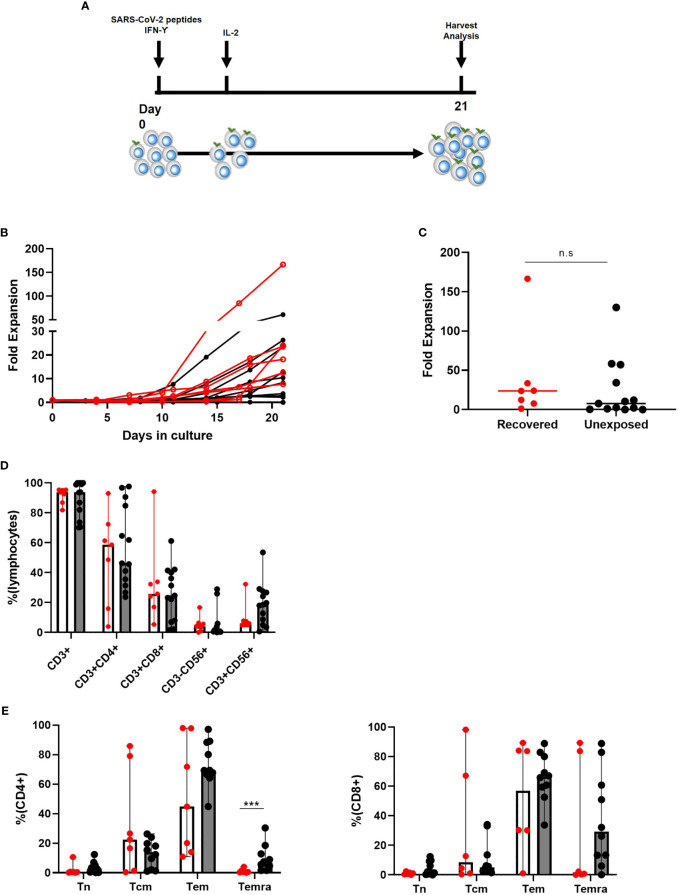
Characteristics of SARS-CoV-2-specific T cells from recovered (n = 7) and unexposed (n = 13) individuals. **(A)** Generation method. **(B, C)** Fold expansion of cells after 21 days of culture (Red indicates COVID-19 recovered individuals; black indicates unexposed individuals). **(D)** Immunophenotypic analysis: COVID19-recovered (red circles with white bar) and unexposed (black circles with gray bars) individuals. **(E)** Memory type of CD4+ and CD8+ T cells. Tn: CD45RA+CD62L+ naive T cells; Tcm: CD45RA−CD62L+ central memory T cells; Tem: CD45RA−CD62L− effector memory T cells; Temra: CD45RA+CD62l− terminally differentiated effector memory T cells; *n.s*., not significant. ***p <.001. The bar indicates median and the error bars indicate range.

We collected either 50 cc of peripheral blood, or one blood volume leukapheresis from both COVID-19 recovered and unexposed donors. Following isolation, PBMCs were stimulated with SARS-CoV-2 S, M, and N peptides along with IFN-γ. We then induced cell proliferation by addition of IL-2 and expanded the cultures for 21 days (**Figure 1A**). While the expansion rate varied among individuals, overall expansion seemed to be more effective in COVID-19 recovered individuals (range: 1.20–166.50, median: 23.7) compared to unexposed individuals (range: 0.0129–130, median: 7.8; **Figures 1B, C**), but the differences were not statistically significant. We investigated the immune cell subsets in the final cell products of COVID-19 recovered and unexposed individuals (**Figure 1D**). The cells showed prominent CD3+ T cell immunophenotype (median: 93.8 and 93.9 in recovered and unexposed, respectively) with the presence of CD3+CD56+ T cells (median: 18.5 and 6.07 in recovered and unexposed, respectively). CD14+ monocytes, CD3-CD56+ natural killer cells, and CD19+ B cells were either absent or less than 5% (**Figure 1C**). There were no significant differences in immunophenotypes of the final cell products between recovered and unexposed individuals. Within CD3+ T cells, we observed slightly higher levels of CD4+ T cells (median 58.6 and 46.3 in recovered and unexposed, respectively) compared to CD8+ T cells (median: 25.8 and 24.7 in recovered and unexposed, respectively). We examined the changes in immunophenotypes before culture, and post-culture on days 4, 7, 14, and 21. We observed a decrease in all immune cell subsets until day 7 of culture, suggesting that cell deaths were not affected by the added SARS-CoV-2 peptides and cytokines (**Supplementary Figure 1**).

Moreover, the memory T cell subtypes of CD4+ and CD8+ in recovered and unexposed individuals showed reduced levels of CD45RA+CD62L+ naive T cells and increased levels of CD45RA+CD62L− central memory and CD45RA−CD62L− effector memory T cells (**Figure 1E**). Final products from COVID-19 recovered individuals showed higher central memory CD4+ and CD8+ T cell levels, compared to unexposed individuals; however, the differences were not significant. Final products from unexposed individuals showed significantly higher levels of CD45RA+CD62L− terminally-differentiated memory CD4+ T cells, but comparable CD8+ T cell levels. There was an overall decrease in naive CD4+ and CD8+ T cells, until there were almost none present on the day of harvest (**Supplementary Figure 1B**). On the other hand, effector memory CD4+ T cells showed a sudden increase during the last week of culture, accompanied by a drop in central memory CD4+ T cell levels. For CD8+ T cells, effector memory and terminally-differentiated effector memory cells showed a steady increase, but central memory CD8+ T cells showed a rapid drop during the last week of culture.

Therefore, expansion of SARS-CoV-2 specific T cells were feasible in both recovered and unexposed individuals and these final T cell products consisted of mainly CD4+ and also CD8+ effector memory T cells with the presence of CD3+CD56+ T cells.

### Expanded SARS-CoV-2 Specific T Cells From Recovered Individuals Showed Higher Antigen-Specificity Against SARS-CoV-2 S, M, and N Proteins

**Figure 2:**
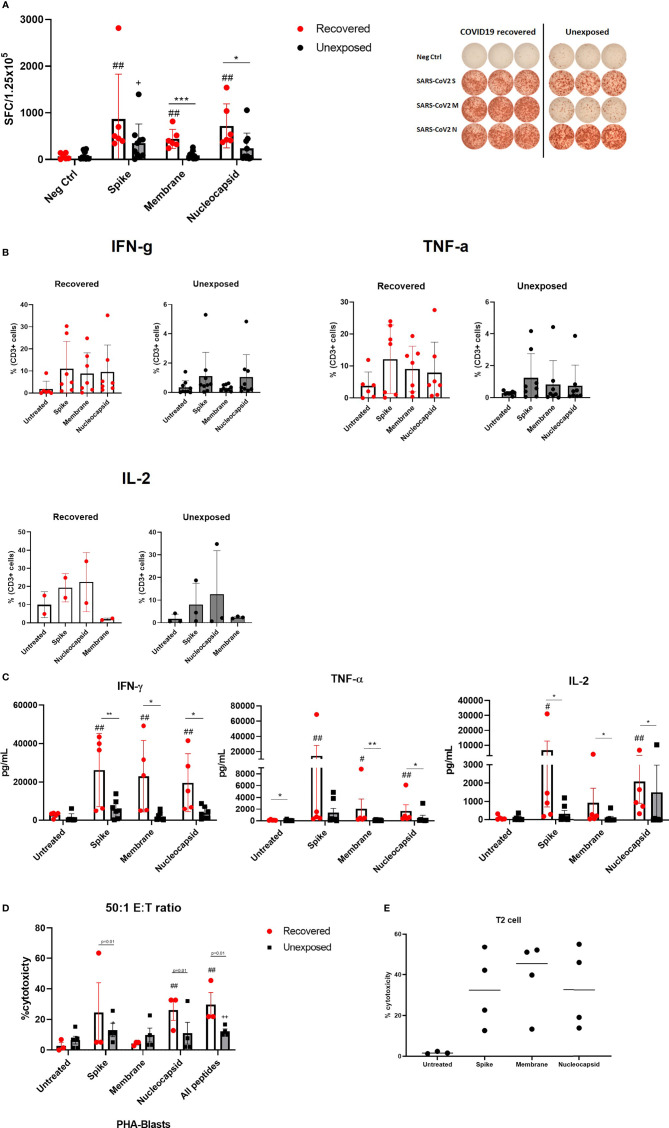
Antigen specificity of SARS-CoV-2-specific T cells for SARS-CoV-2 proteins. **(A)** IFN-γ ELISPOT assay against S, M, and N peptide mixtures covered viral proteins in COVID19 recovered (n=6, red circles with white bar) and unexposed (n=9, black circles with gray bar) individuals. The number of spot-forming cells per 1.25 x 10^5^ cells are shown. Neg Ctrl indicates negative controls, in which the cells were treated with fresh complete medium only. SARS-CoV-2-specific T cells were treated with S, M, or N peptide mixtures for 24 hours. Right panel shows a representative ELISPOT result between COVID-recovered and unexposed individual **(B)** Flow cytometric analysis of CD3+ T cell intracellular cytokine production from recovered (n=6) and unexposed (n=9) individuals. **(C)** Supernatant multiplex assays to measure cytokine production from recovered (n=6, red circles with white bar) and unexposed (n=9, black circles with gray bar) individuals. Untreated group was the negative control group. **(D)** Specific cytotoxic activity of SARS-CoV-2 specific T cells on autologous non-pulsed (untreated) and SARS-CoV-2 peptide pulsed PHA-induced blasts. Cells were co-cultured at a 50:1 effector to target ratio for 4 hours, and cell deaths were determined using flow cytometry. Cytotoxicity of SARS-CoV-2 T cells produced from recovered (n=3, red circles with white bar) and unexposed (n=5, black circles with gray bar) individuals were compared. **(E)** Cytotoxic effect against SARS-CoV_2 peptide pulse T2 cell line was evaluated. SARS-CoV-2 specific T cells (n=4) were co-cultured at 50:1 effector to target ratio overnight and cell death was determined using flow cytometry. Each circle represents a donor. Statistical comparisons between unexposed and recovered individual groups are indicated as follows *p <.05; **p <.01; ***p <.001. Statistical comparisons between recovered negative control and peptide mixture treated groups are indicated as follows ^#^p<.05; ^##^p<.01. Statistical comparisons between unexposed negative control and peptide mixture treated groups are indicated as follows ^+^p<.05; ^++^p<.01.

Next, we determined whether the expanded cells were specific to SARS-CoV-2 S, M, and N proteins by detecting anti-viral cytokine production following antigenic stimulation. We performed ELISPOT assays to determine IFN-γ secreting cells against each SARS-CoV-2 protein. All but two unexposed donors showed IFN-γ secreting cells against at least one protein among three of SARS-CoV-2 S, M, and N proteins. In other words, 18 out of 20 SARS-CoV-2 specific T cell products showed IFN- γ production against one or more SARS-CoV-2 proteins. The numbers of IFN-γ secreting cells against S-glycoprotein were similar between COVID-19 recovered and unexposed individuals. However, the numbers of IFN-γ secreting cells against M and N proteins were significantly higher in COVID-19 recovered individuals (**Figure 2A**). Prior to culture, the numbers of IFN-γ secreting cells were higher in recovered individuals, whereas almost none were detected in unexposed individuals (**Supplementary Figure 2A**). However, intracellular cytokine secreting assay by flow cytometry showed that the antigen-specific cytokine production between recovered and unexposed individuals were almost similar with no significant differences prior to culture (**Supplementary Figure 2B**). After 21 days of culture, antigen-specific cytokine production including IFN-γ, TNF- α, and IL-2 were expanded with significantly elevated levels in the recovered group (**Figure 2B**). CD4+, CD8+, and CD3+CD56+ T cells were mainly responsible for IFN-γ and TNF-α secretion, whereas CD8+ T cells and a proportion of CD3-CD56+ were the source of IL-2 production (**Supplementary Figure 2C**). Similar to the ELISPOT results, IFN-γ secreting cells were increased against S and N peptide mixtures. Moreover, TNF-α cells and IL-2 secreting cells were responsive to S and N restimulation. The cytokine production in the supernatants was similar to the previous results (**Figure 2C**), but IFN-γ production against M peptide stimulation was higher in the culture supernatant. The levels of cytokine production in response to antigen re-stimulation was significantly higher in COVID-19 recovered individuals compared to unexposed individuals’ SARS-CoV-2 T cell product.

To test for direct cytotoxic effects, we used SARS-CoV-2 peptide pulsed autologous PHA-blasts as an alternative to SARS-CoV-2 infected cells. We observed higher cytotoxicity with SARS-CoV-2 peptide pulsed PHA-blasts compared to unpulsed PHA-blasts (**Figure 2D**). There was a slight increase in cytotoxicity when all three peptides were pulsed in comparison to single peptide pulsing. While the trend indicated that the cytotoxic effects of SARS-CoV-2 specific T cells from recovered individuals tend to be higher than those from unexposed individuals, a broad variation of cytotoxicity between donors still exist for direct comparison. In addition, we co-cultured HLA-A2 expressing SARS-CoV-2 specific T cells with SARS-CoV-2 peptide-pulsed T2 cell line (**Figure 2E**) demonstrating high anti-viral cytotoxic effects. While the direct cytotoxic effects of SARS-CoV-2 specific T cells differed between each individual, granzyme B and perforin secreting cells were consistently upregulated in the cells expanded from both recovered and unexposed individuals. CD8+ T cells were the major source of granzyme B and perforin prior to culture; however, All major immune subsets of the final products were able to produce these cytotoxic molecules (**Supplementary Figures 3A, B**). Moreover, granzyme B and perforin levels were produced at similar levels in the final cell products from both unexposed and COVID-19 recovered individuals (**Supplementary Figure 3C**).

Our observations indicate that SARS-CoV-2 specific T cells show antigen specificity through cytokine production in response to antigenic restimulation, direct cytotoxic effects against antigen expressing target cells and also the secretion of cytotoxic molecules. However, SARS-CoV-2 specific T cells cultured from recovered individuals had a stronger antigen-specific cytokine response.

### SARS-CoV-2 Specific T Cells Showed Activated Markers Associated With Immunogenicity

**Figure 3:**
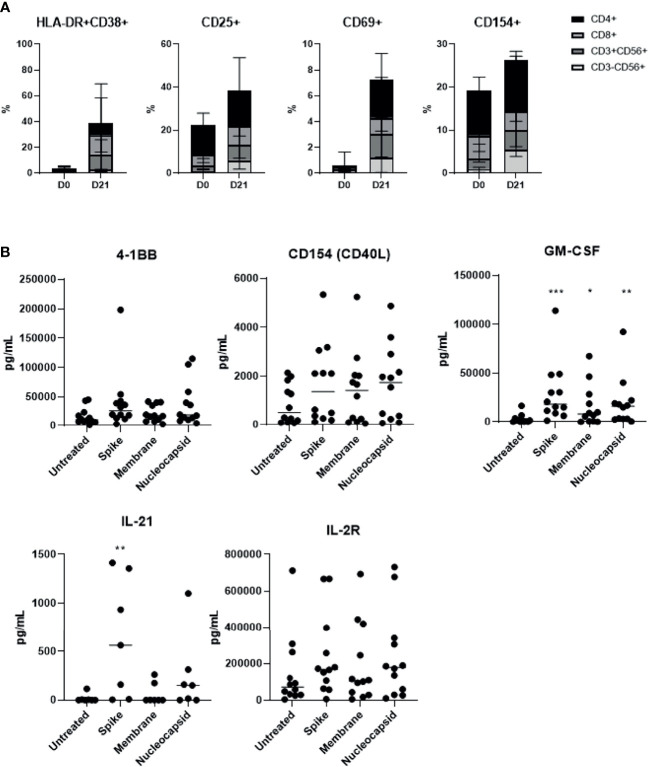
SARS-CoV-2-specific T cells showed high activation marker levels, which were further up-regulated in response to antigenic stimulation (n=15, 6 recovered and 9 unexposed individuals). **(A)** HLA−DR+CD38+, CD25+, CD69+ and CD154+ for all lymphocyte subsets were measured prior to and after 21 days of culture. **(B)** SARS-CoV-2-specific T cells were treated with S, M, or N peptide mixtures for at least 18 hours, and the supernatant was collected to measure T-cell activation-related cytokine production from 5 unexposed and 7 recovered individuals. *p <.05; **p <.01; ***p <.001.

We determined activation markers of the final cell products and were able to observe an activated phenotype with up-regulated levels of HLA-DR+CD38+, CD25, CD69, and CD154 in all three major T cell subsets, following 21 days of culture (**Figure 3A**). These changes following SARS-CoV-2 peptide antigenic stimulation were minimal and not significant suggesting the activated nature of the product. Furthermore, we examined cytokines involved in T cell activation, such as 4-1BB, CD40L (CD154), and IL-2R, and those that promote immunogenicity, such as GM-CSF and IL-21 (**Figure 3B**). Most of these cytokines increased following S-glycoprotein stimulation. The levels of 4-1BB remained upregulated, indicating overall T cell activation within the products. Slightly higher activated and immunogenic phenotype was observed from the final SARS-CoV-2 specific T cell product produced from COVID-19 recovered individuals than unexposed individuals; however, there was no statistical significance (**Supplementary Figure 4**). The results suggest that the SARS-CoV-2 specific T cells regardless of donor origin possess a memory T cell phenotype that is associated with the activated markers and moreover, produce immunogenic cytokines that may potentially induce anti-SARS-CoV-2 immunity.

### Third-Party SARS-CoV-2-Specific T Cells Did Not Show Alloreactivity in a Mixed Lymphocyte Reaction

**Figure 4:**
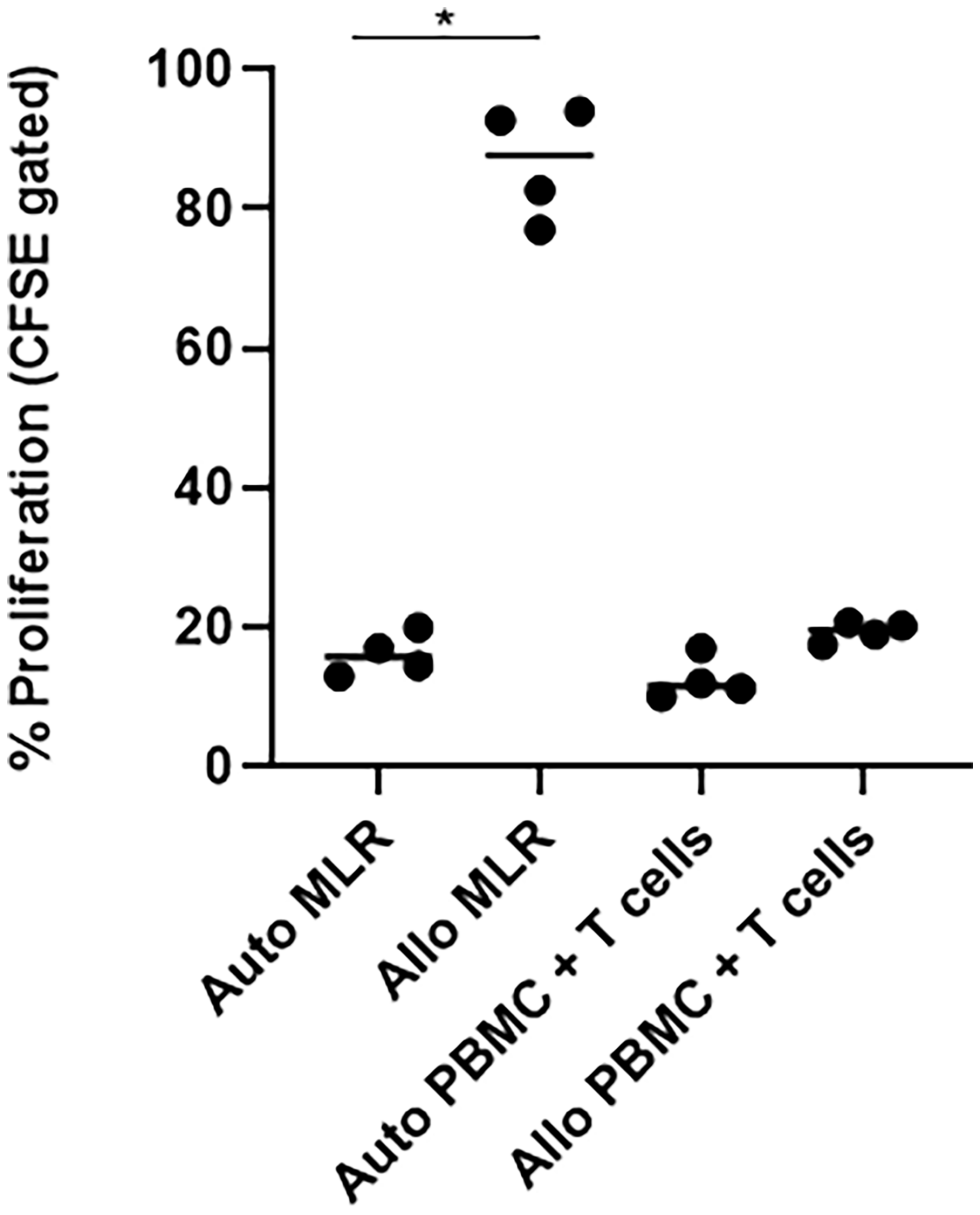
SARS-CoV-2 specific T cells (n=4) did not show alloreactivity against HLA-unmatched PBMCs. T cells, SARS-CoV-2-specific T cells; auto, autologous; allo, allogeneic; MLR, mixed lymphocyte reaction; PBMC, peripheral blood mononuclear cells. Autologous mixed lymphocyte group was used as negative control, while allogeneic mixed lymphocyte group was used as positive control. *p <.05.

We assessed the allogeneicity of SARS-CoV-2 specific T cells against autologous and HLA-mismatched allogenic PBMCs by CFSE proliferation assay. As a control, we co-cultured HLA-mismatched PBMCs from two different donors, or CFSE-labelled and un-labelled PBMCs from the same donor for five days (**Figure 4**). The CFSE-labelled responder cells showed active proliferation in the allogeneic condition, suggesting alloreactive T cells against the respective stimulating antigen-presenting cells. In contrast, when we co-cultured SARS-CoV-2 specific T cells against HLA-mismatched allogeneic PBMCs, the proliferation was minimal compared to the allogeneic control, and similar to the autologous control, suggesting that the ex-vivo expanded SARS-CoV-2 specific T cells did not show alloreactivity.

### SARS-CoV-2 Specific T Cells Contained Regulatory T Cells and Expressed Inhibitory Markers

**Figure 5:**
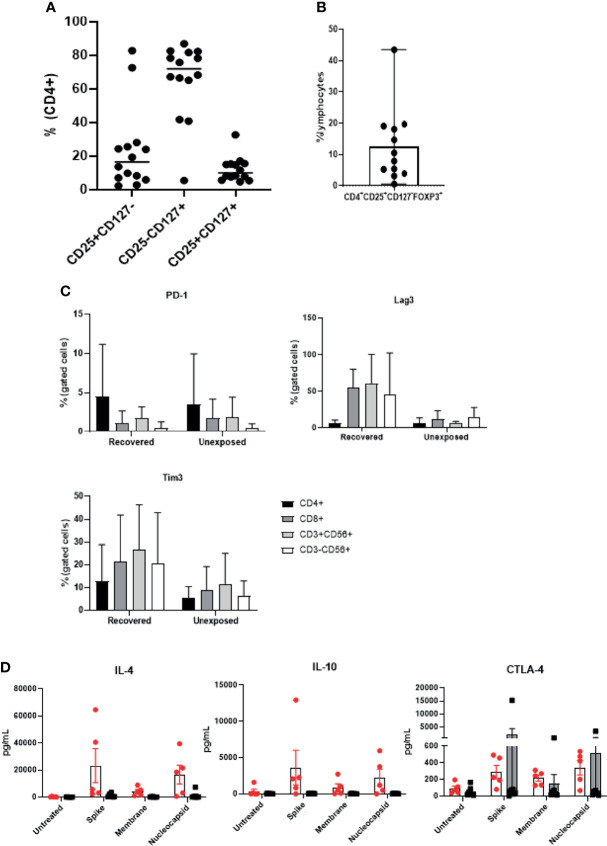
Expanded cell products show presence of Tregs and inhibitory markers. (n=14) **(A)** The percentages of CD4+CD25^high^CD127^low^, CD4+CD25^low^CD127^high^; Tcons, and CD4+CD25^high^CD127^high^ cells are indicated from 9 unexposed and 5 recovered individuals) **(B)** Percentage of CD4+CD25^high^CD127^low^ Foxp3+ Treg ratio was determined (n=12). Each circle represents a cell product produced from a single donor. **(C)** Flow cytometric analysis of immune checkpoint marker (PD-1+, Tim-3+, and LAG-3+) expression of each lymphocyte subset was measured from recovered (n=6) and unexposed (n=9) individuals. **(D)** SARS-CoV-2-specific T cells were treated with S, M, or N peptide mixtures for at least 18 hours, and the supernatant from recovered (n=6, red circles with white bar) and unexposed (n=9, black circles with gray bar) individuals was collected to measure anti-inflammatory cytokine production.

With the observation that SARS-CoV-2 specific T cells showed high expression of CD4 and CD25, we examined the presence of CD4+CD25+CD127− cells within the final products (**Figure 5A**). CD4+CD25+CD127 levels (median: 6.365; range: 0.59–66.72) varied between donors, but there were no significant differences between the donor groups (**Supplementary Figure 5A**). The majority of CD4+ T cells were CD4+CD25−CD127+ cells (Tcons). Majority of the CD4+CD25+CD127- cells were regulatory T cells (Tregs) confirmed by Foxp3 expression (**Figure 5B**). Next, we examined three representative inhibitory markers: PD-1, Tim-3, and LAG-3. All three markers were upregulated following 21 days of culture (**Supplementary Figure 5B**). Although PD-1 expression was similar in both donor groups, Tim-3 and LAG-3 levels were up-regulated in all four subsets especially in the products of recovered individuals (**Figure 5C**). We investigated further potentially relevant anti-inflammatory cytokines. Similarly, IL-4 and IL-10 were found to be increased in response to antigenic stimulation especially in the products of COVID-19 recovered individuals (**Figure 5D**). Since we did not confirm all relevant exhaustion markers and transcription factors, it is unclear whether these cells were strongly activated, exhausted, or possessed inhibitory functions, and further studies will be required for better characterization of these cells.

## Discussion

The emergence of the COVID-19 pandemic in 2019 has led to global efforts to develop effective vaccines and treatments. Unlike previous viral outbreaks, SARS-CoV-2 is known to be highly contagious and can spread at unprecedented rates. While the vaccination process has been accelerated worldwide to achieve herd immunity, there has been little progress in COVID-19 treatment options (23).

Acute respiratory distress syndrome (ARDS), respiratory failure, and cytokine storm syndrome due to a hyper-inflammatory cytokine profile, are the leading causes of mortality in COVID-19 (24, 25). Adjuvant treatments, such as corticosteroids and immunomodulators are being used to alleviate hyper-inflammation. Evidence suggests that both dexamethasone (26, 27) and anti-IL-6 antibodies (28, 29) can improve COVID-19 survival and mortality rates. These approaches treat COVID-19 complications instead of eliminating viral replication or virus-infected cells. In fact, these anti-inflammatory drugs may adversely affect the anti-viral immune responses. To combat SARS-CoV-2 infections directly, genetically modified neutralizing antibodies have been developed that bind to the receptor-binding domains of the S protein of SARS-CoV-2, block S-protein attachment to human ACE2 receptors, and inhibit virus entry into cells (30, 31). However, antibodies are only effective when viruses are rapidly replicating within the body, which is usually one week after infection. Patients with severe ARDS or pneumonia do not respond well to antibody treatment, as the viruses are not present in the blood stream (14). Experience with human coronaviruses, including MERS-CoV and SARS-CoV-2, suggests that antibody titers decline over time (15). Therefore, once viral infection is established, cellular immunity is the final and the most important line of defense.

In our study, we suggest a treatment approach that relies on the most fundamental concept in viral immunology: adaptive immunity, specifically T cells. The main purpose of SARS-CoV-2-specific T cell therapy is to reverse actively and restore inadequate T cell responses, to improve viral control while minimizing excessive inflammatory responses. Our results demonstrated that manufacturing antigen-specific T cells from either COVID-19 recovered or unexposed individuals is feasible, and reproducible within 21 days. Our ex vivo expanded SARS-CoV-2 antigen-specific memory T cell products were polyclonal, containing both CD4+, CD8+, and CD56+ T cells (**Figure 1**), with antigen-specific IFN-γ, TNF-α, and IL-2 production, leading to direct cytotoxic effects against target cells (**Figure 2**). These T cells showed a functionally-activated phenotype, producing cytokines that may participate in recruitment of immune cells and induce anti-viral immunity (**Figure 3**). However, they did not induce an allogeneic response against HLA-mismatched target cells, suggesting a possibility for off-the-shelf third-party products. A proportion of the cell products consisted of CD4+CD25^high^CD127^low^ Foxp3-expressing Tregs (**Figure 5**). However, because we did not study the functionality of these Tregs, further studies need to confirm whether these cells do exhibit anti-inflammatory effects. In addition, Tim-3 and Lag-3 in particular were upregulated in the products from COVID-19 recovered individuals. The co-expression of the two markers associated with the presence of Tregs and secretion of IL-4 and IL-10 seem to suggest immunosuppressive capabilities. Whether these marker indicate functionality, hyperactivation or exhaustion remains to be elucidated and we hope to address this in future studies. Nonetheless, the heterogenous characteristics of our SARS-CoV-2-specific T cell products were unique and important for COVID-19, which requires varying immunological strategies.

SARS-CoV-2 specific T cells as a therapeutic option have been previously reported (**Table 3**), and a few clinical trials are registered and open for recruitment (**Table 4**). The majority of these clinical trials concentrate on treating patients with severe disease, or at a high risk for developing severe disease, such as cancer patients and older adults. There are two main methods for SARS-CoV-2 T cell generation: the ex-vivo expansion method, and direct selection of T cells. Each method has its advantages and disadvantages. Direct selection of T cells responding to SARS-CoV-2, or selection of memory T cells is convenient in emergency situations. However, it is not scalable because the final cell product numbers are relatively low. Ex-vivo expansion methods are more time-consuming and require more experience. However, mass production is possible and, depending on the method used, has the potential to treat hundreds to thousands of patients, using only a few donors. Furthermore, establishment of a mini bank for T cells with common HLA types may allow third-party off-the-shelf use of SARS-CoV-2-specific T cells (16). We have produced clinical-grade SARS-COV-2-specific T cells at a GMP facility. While the expansion rates showed donor variabilities, between 40 and 713 doses could be produced, considering a dose of 2x10^7^ cells/m^2^ for an average adult (**Table 5**).

**Table 3:** Published pre-clinical studies on SARS-CoV-2 specific T cells.

Country	Donor	Generation method	Main Immunophenotypical characteristics of final cell product	Reference
United States	COVID-19 convalescent or vaccinated individual	*Ex-Vivo* expansion	–	(21)
Spain	COVID-19 convalescent or unexposed individual	*Ex-Vivo* expansion	CD3+CD4+	(8)
Spain	COVID-19 convalescent individual	Separation of memory T cells	CD3+CD4+CD8+ CD45RA-	(11)
Germany	COVID-19 convalescent individual	Separation of IFN-γ+ producing cells responding to SARS-COV-2 peptides followed by *ex-Vivo* expansion	CD3+CD4+CD8+ CD45RA- IFN-γ+	(10)
United States	COVID-19 convalescent or unexposed individual	*Ex-Vivo* expansion	CD4 dominant T cells	(9)
United States	COVID-19 convalescent or unexposed individual	*Ex-Vivo* expansion	NR3C1 gene transduced T cells	(32)
Singapore	COVID-19 patients	Separation of IFN-γ+ producing cells responding to SARS-COV-2 peptides	CD3+CD4+,CD8+,CD56+ CD45RA- IFN-γ+	(33)

**Table 4:** Registered clinical trials using SARS-CoV-2 T cells to treat COVID-19.

Country	Phase	# of patients	Target Patients	Cell Dose	Generation Method	Status	Clinical trial identifier number
Singapore	I/II	18	Severe COVID-19	Unknown	Cell Separation	Recruiting	NCT04457726
Germany	I/II	51	Severe COVID-19	1000-5000 cells/kg	Cell Separation	Not yet recruiting	NCT0476186
United States	I	16	SARS-COV-2 infected cancer patients	Unknown	*Ex-Vivo* expansion	Recruiting	NCT04742595
United States	I/II	58	Severe COVID-19	1, 2, 4x10^7^/m^2^	*Ex-Vivo* expansion	Recruiting	NCT04401410
United States	I	24	Elderly patients with severe COVID-19	Unknown	Unknown	Not yet recruiting	NCT04765449
Spain	I/II	58	Severe COVID-19	Unknown (dose-escalation is included)	Cell Separation	Recruiting	NCT04578210

**Table 5:** GMP production of SARS-CoV-2 specific T cells.

Donor #	Starting Material	Fold Expansion	Theoretical number of doses that can be produced^*^
16	Leukapheresis	3.5	294 doses
17	Leukapheresis	1.2	713 doses
18	Whole Blood	8.4	47 doses
19	Whole Blood	10.4	40 doses
20	Leukapheresis	7.8	265 doses

*One dose was considered as 2x10^7^/m^2^ body surface area. Body surface area of an average adult male 1.9m^2^ was used for calculation.

Because antigen-specific cytokine production tends to be higher in recovered individuals, the use of T cells generated from recovered individuals may be more useful as a therapeutic drug. However, cells generated from unexposed individuals may still be applied as a personalized T cell vaccines especially for immunocompromised individuals and those who are at a high risk for vaccine adverse effects. While, our results also showed comparable expansion from unexposed individuals, the antigen-specific cytokine production and cytotoxicity remained low compared to cells produced from COVID-19 recovered individuals, possibly due to cross-reactivity with other common corona viruses (7). We hypothesized that once the autologous SARS-CoV-2 T cells are infused into a healthy individual, the cells would circulate in the bloodstream and lymphatics to induce a vaccine-like anti-viral immune response. Because our products were predominantly CD4+ T cells, which produced IL-21, they may potentially interact with B cells to promote production of neutralizing antibodies. However, this remains to be elucidated and requires investigation in a clinical trial.

More recently, with on-going variant outbreaks due to mutations in the SARS-CoV-2 gene, therapeutic options that specifically target the spike glycoprotein have become ineffective. Based on our results, ex vivo expanded SARS-CoV-2 specific T cells can target three major SARS-CoV-2 peptides including membrane and nucleocapsid proteins. This implies that cell therapy can be a resistant and effective approach against future SARS-CoV-2 mutations.

Our study had some limitations, which may need to be addressed in future studies. Further functional analyses of the produced cells need to be performed. The cells exhibited a heterogenous population that included antigen-specific, as well as antigen-independent activated cells. While our results showed antigen-specific cytokine production, the cytotoxicity effects remained moderate at high effector to target ratios, possibly due to CD4 dominance of the cell products. Further studies are required to clarify the mechanism of action of each cell population. For instance, CD4 and CD8 cells may be isolated to determine their individual cytotoxicities. In addition, due to limited access to MHC class I and II pentamers at the time of the study, we could not directly enumerate antigen-specific T cells. Sorting antigen-specific and non-specific cells may help determine their roles in viral control. Detailed phenotypical analyses are also needed, especially for CD4+ T cells, as our observations have suggested the presence of follicular helper T cells within the product. Moreover, Tregs and the inhibitory markers induced in the final product need to be analyzed to determine whether they demonstrated functional exhaustion or immunosuppressive capacity. Our study also indicated the possibility of inducing SARS-CoV-2-specific T cells from SARS-CoV-2 unexposed donors. Larger-scale studies with more unexposed donors are required to investigate this possibility further. With an increasing number of vaccinated individuals, it will be intriguing to see whether vaccinations affect the manufacture of SARS-CoV-2-specific T cells.

The pivotal role of T-cell immunity in viral infections is well known, and has been used in clinical practice for over 20 years (17). Adoptive transfer of VSTs has proven to be safe, with minimal adverse events (18). Advances in manufacturing methods have significantly reduced production time (19), and commercialization is possible by third-party VST banks (16, 20). Currently, there are no effective treatments for hospitalized COVID-19 patients. Therefore, efforts must be directed toward developing newer therapeutic approaches.

In conclusion, we believe that development of treatment strategies for hospitalized COVID-19 patients is a matter of some urgency. SARS-CoV-2-specific T cells are a promising approach for treating COVID-19, one that can directly eliminate infected cells, regulate hyper-inflammatory responses, and provide long-term anti-viral immunity.

## Data Availability Statement

The original contributions presented in the study are included in the article/**Supplementary Material**. Further inquiries can be directed to the corresponding author.

## Ethics Statement

The studies involving human participants were reviewed and approved by Institutional Review Board of Seoul St. Mary’s Hospital. The patients/participants provided their written informed consent to participate in this study.

## Author Contributions

SGC designed the study. NK, J-ML, E-JO, and K-II collected data. S-GC, NK, J-ML, E-JO, and K-II analyzed and interpreted data. NK, J-ML, E-JO, K-II, and S-GC wrote and revised the manuscript. NK, J-ML, E-JO, K-II, and S-GC approved the final version of the manuscript. All authors contributed to the article and approved the submitted version.

## Funding

This study was supported by Lucas Bio Co., Ltd.

## Conflict of Interest

Authors NK, K-II and S-GC were employed by company LucasBio Co., Ltd.

The remaining authors declare that the research was conducted in the absence of any commercial or financial relationships that could be construed as a potential conflict of interest.This study received funding from Lucas Bio Co., Ltd.

The funder had the following involvement with the study: study design, collection, analysis, interpretation and the writing of this article

## Publisher’s Note

All claims expressed in this article are solely those of the authors and do not necessarily represent those of their affiliated organizations, or those of the publisher, the editors and the reviewers. Any product that may be evaluated in this article, or claim that may be made by its manufacturer, is not guaranteed or endorsed by the publisher.

## Supplementary Material

Supplementary Figure 1 Kinetics of immune cell subsets during 21 days of culture (n=5). Changes in **(A)** immunophenotype, **(B)** CD4+, and **(C)** CD8+ memory T cells during culture of SARS-CoV-2-specific T cells from 3 unexposed and 2 recovered individuals.

Supplementary Figure 2 Antigen specificity of SARS-CoV-2 specific T cells prior to culture from unexposed (n=3) and recovered (n=2) individuals through **(A)** ELISPOT assay and **(B)** intracellular cytokine staining using flow cytometry. **(C)** Following 21 days of culture, different cell subsets including CD8+, CD4+, CD3+CD56+ and CD3-CD56+ produced antigen specific IFN-ϒ TNF-α and IL-2 in response to peptide re-stimulation. The difference in cytokine production levels are shown between recovered (n= 7) and unexposed (n=8) individuals.

Supplementary Figure 3 SARS-CoV-2 specific T cells show robust production of cytotoxic molecules, granzyme B and perforin (n=15). **(A)** Ex-vivo culture induces granzyme B and perforin in SARS-CoV-2-specific T cells. **(B)** SARS-CoV-2 specific T cells (n=15) were treated with S, M, or N peptide mixtures for at least 18 hours. Stimulated cells were collected for flow cytometric analysis of granzyme-b-producing, and granzyme-b- and perforin-producing cells of each lymphocyte subset. The levels remain unchanged after antigenic re-stimulation. **(C)** Cell culture supernatants from recovered (n=6, red circles with white bar) and unexposed (n=9, black circles with gray bar) individuals after re-stimulation were collected for granzyme B and perforin measurement. Results represent data from 3 unexposed and 2 recovered individuals. *p <.05; **p <.01; *p <.001.

Supplementary Figure 4 Activation markers and related cytokine production of SARS-CoV-2 specific T cells between unexposed and recovered individuals (n=15). **(A)** HLA−DR+CD38+, CD25+, CD69+ and CD154+ for all lymphocyte subsets were measured prior to, and after 21 days of culture. **(B)** SARS-CoV-2-specific T cells from recovered (n=6, red circles with white bar) and unexposed (n=9, black circles with gray bar) individuals were treated with S, M, or N peptide mixtures for at least 18 hours, and the supernatant was collected to measure T-cell activation-related cytokine production. Results represent data from 9 unexposed and 6 recovered individuals.

Supplementary Figure 5
**(A)** The percentages of CD4+CD25^high^CD127^low^, CD4+CD25^low^CD127^high^; Tcons, and CD4+CD25^high^CD127^high^ cells were compared between recovered (n=5) and unexposed (n=9) individuals. **(B)** Inhibitory markers including PD-1, Tim-3, and LAG-3 were upregulated following cell culture in each lymphocyte subset.
