# Supplementary figures and images for: Off-the-Shelf Partial HLA Matching SARS-CoV-2 Antigen Specific T Cell Therapy: A New Possibility for COVID-19 Treatment
Source: Front Immunol. 2021 Dec 23;12:751869. doi: 10.3389/fimmu.2021.751869 (PMC8733616; doi:10.3389/fimmu.2021.751869)

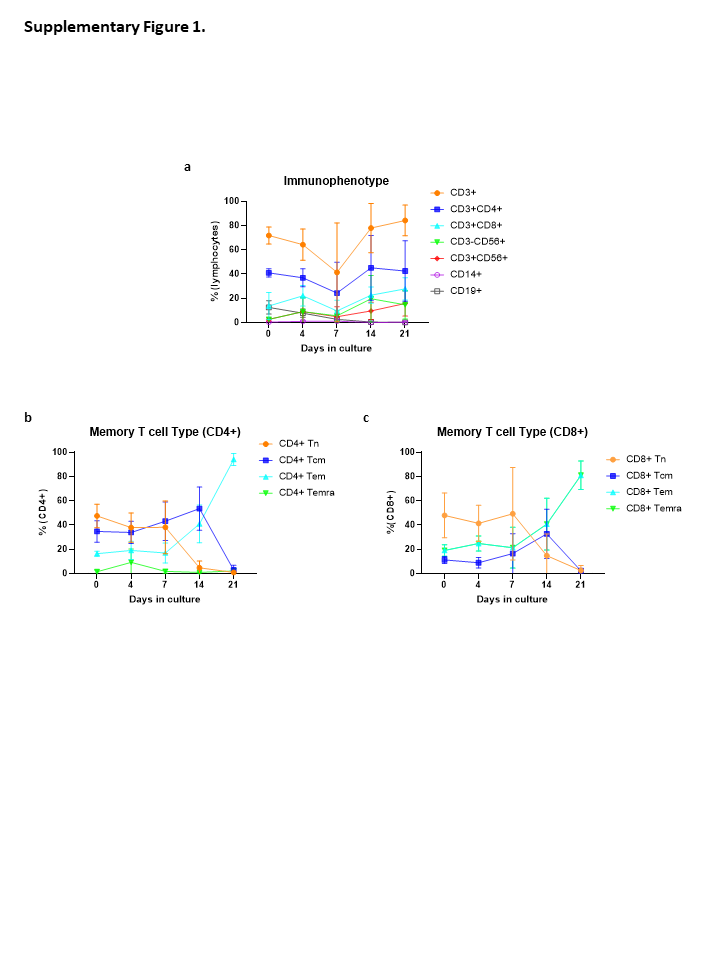

Supplement: Supplementary Figure 1 — Kinetics of immune cell subsets during 21 days of culture (n=5). Changes in (A) immunophenotype, (B) CD4+, and (C) CD8+ memory T cells during culture of SARS-CoV-2-specific T cells from 3 unexposed and 2 recovered individuals. [file Image_1.tif]

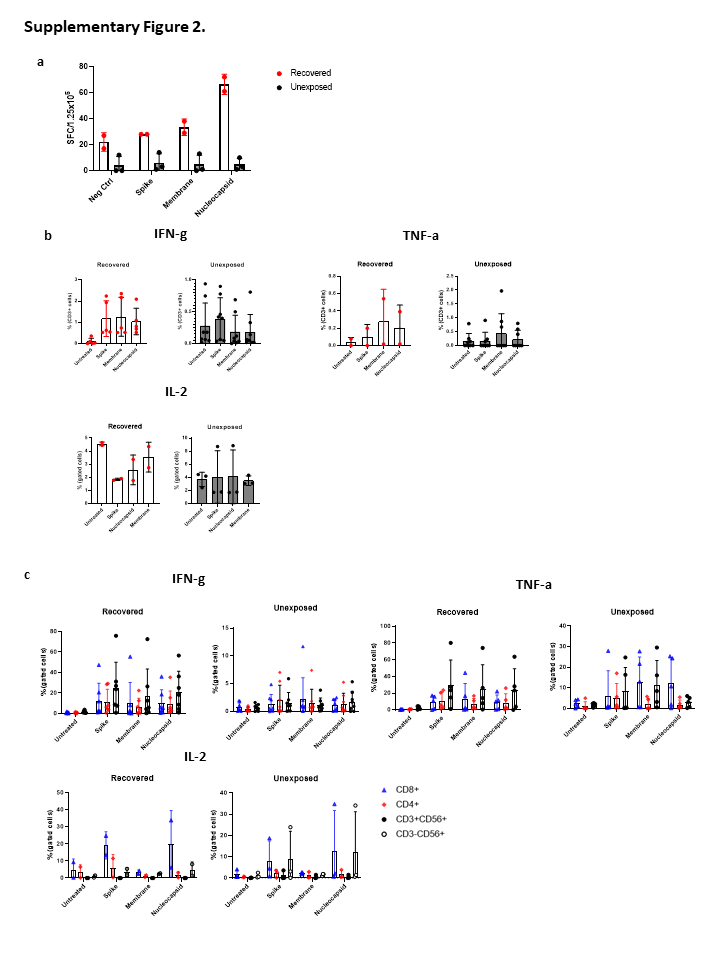

Supplement: Supplementary Figure 2 — Antigen specificity of SARS-CoV-2 specific T cells prior to culture from unexposed (n=3) and recovered (n=2) individuals through (A) ELISPOT assay and (B) intracellular cytokine staining using flow cytometry. (C) Following 21 days of culture, different cell subsets including CD8+, CD4+, CD3+CD56+ and CD3-CD56+ produced antigen specific IFN-ϒ TNF-α and IL-2 in response to peptide re-stimulation. The difference in cytokine production levels are shown between recovered (n= 7) and unexposed (n=8) individuals. [file Image_2.tif]

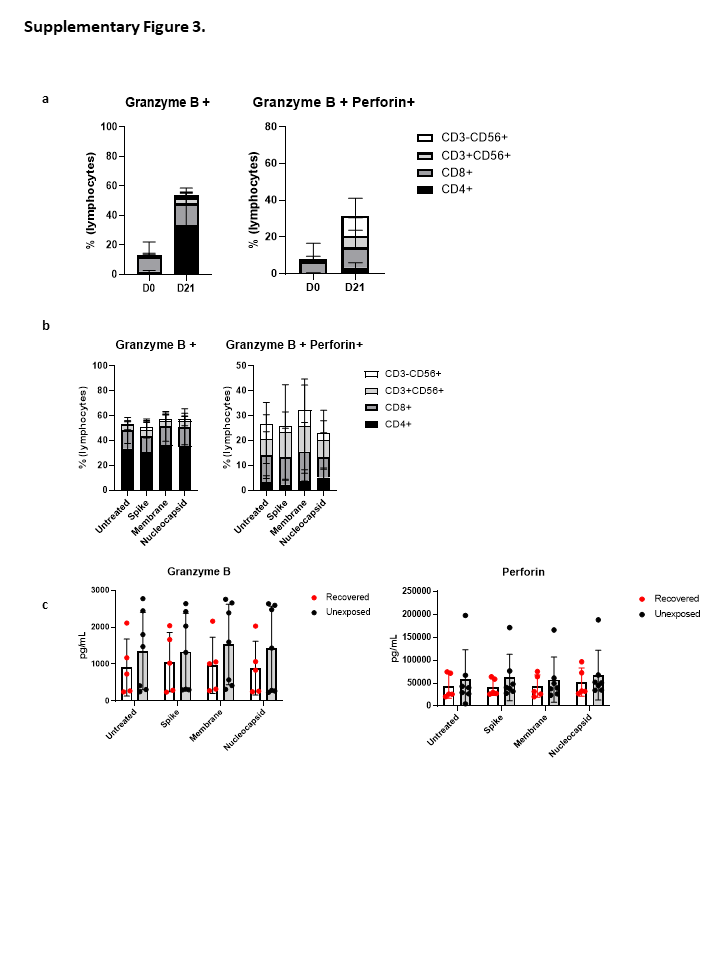

Supplement: Supplementary Figure 3 — SARS-CoV-2 specific T cells show robust production of cytotoxic molecules, granzyme B and perforin (n=15). (A) Ex-vivo culture induces granzyme B and perforin in SARS-CoV-2-specific T cells. (B) SARS-CoV-2 specific T cells (n=15) were treated with S, M, or N peptide mixtures for at least 18 hours. Stimulated cells were collected for flow cytometric analysis of granzyme-b-producing, and granzyme-b- and perforin-producing cells of each lymphocyte subset. The levels remain unchanged after antigenic re-stimulation. (C) Cell culture supernatants from recovered (n=6, red circles with white bar) and unexposed (n=9, black circles with gray bar) individuals after re-stimulation were collected for granzyme B and perforin measurement. Results represent data from 3 unexposed and 2 recovered individuals. *p <.05; **p <.01; *p <.001. [file Image_3.tif]

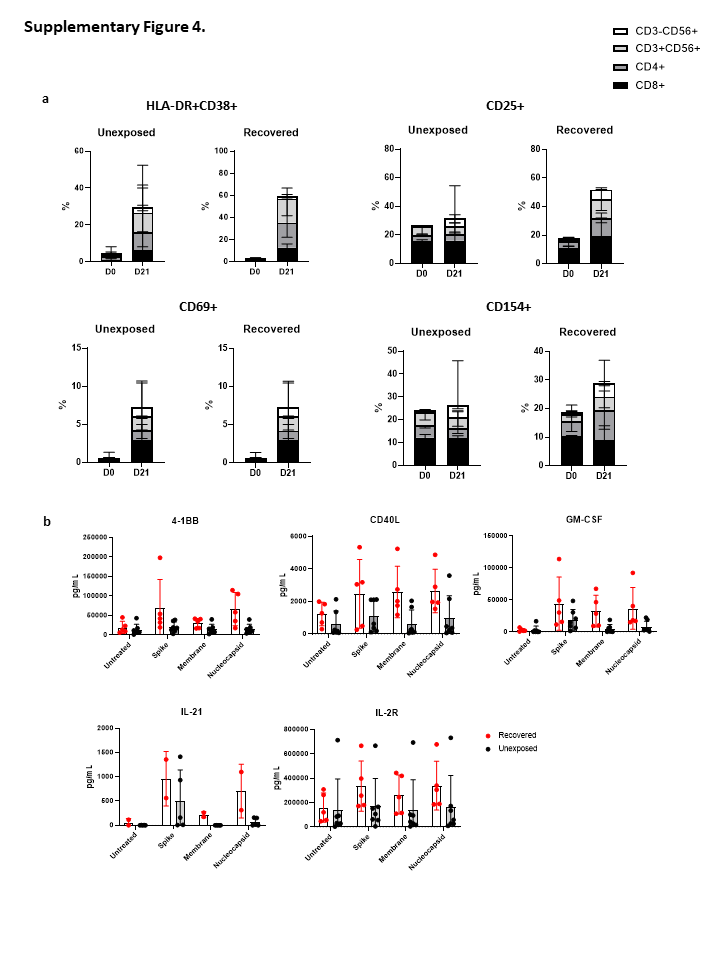

Supplement: Supplementary Figure 4 — Activation markers and related cytokine production of SARS-CoV-2 specific T cells between unexposed and recovered individuals (n=15). (A) HLA−DR+CD38+, CD25+, CD69+ and CD154+ for all lymphocyte subsets were measured prior to, and after 21 days of culture. (B) SARS-CoV-2-specific T cells from recovered (n=6, red circles with white bar) and unexposed (n=9, black circles with gray bar) individuals were treated with S, M, or N peptide mixtures for at least 18 hours, and the supernatant was collected to measure T-cell activation-related cytokine production. Results represent data from 9 unexposed and 6 recovered individuals. [file Image_4.tif]

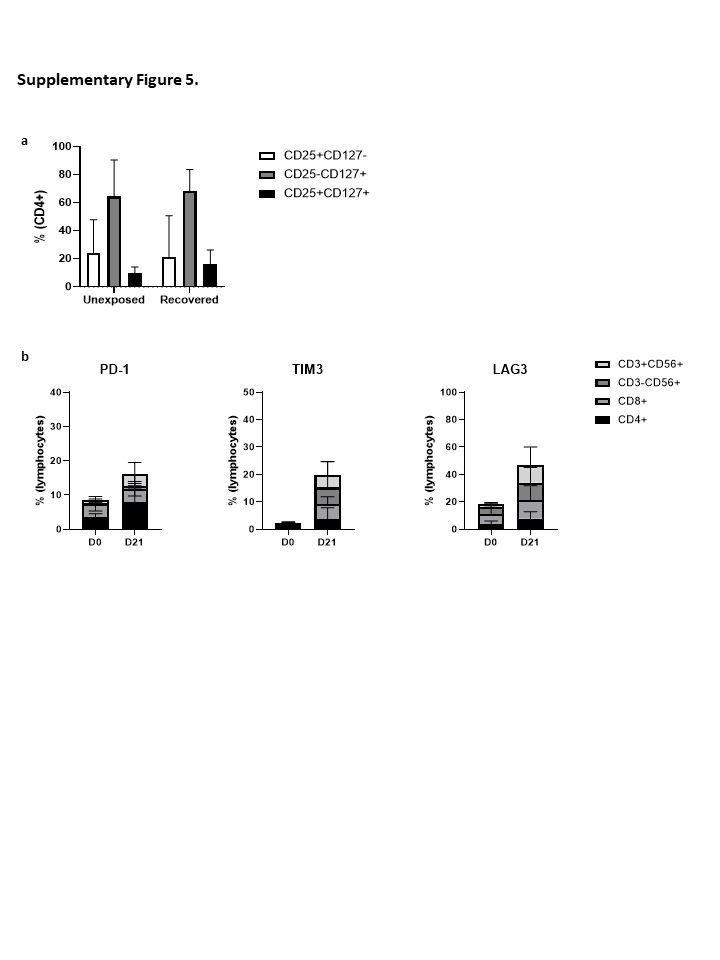

Supplement: Supplementary Figure 5 — (A) The percentages of CD4+CD25highCD127low, CD4+CD25lowCD127high; Tcons, and CD4+CD25highCD127high cells were compared between recovered (n=5) and unexposed (n=9) individuals. (B) Inhibitory markers including PD-1, Tim-3, and LAG-3 were upregulated following cell culture in each lymphocyte subset. [file Image_5.tif]
